# Eco-Friendly Blends of Recycled PET Copolymers with PLLA and Their Composites with Chopped Flax Fibres

**DOI:** 10.3390/polym15143004

**Published:** 2023-07-10

**Authors:** Martial Aimé Kuété, Pascal Van Velthem, Wael Ballout, Nathan Klavzer, Bernard Nysten, Maurice Kor Ndikontar, Thomas Pardoen, Christian Bailly

**Affiliations:** 1 Institute of Condensed Matter and Nanosciences—Bio & Soft Matter (IMCN/BSMA), UCLouvain, 1348 Louvain-la-Neuve, Belgium; martial.kuete@uclouvain.be (M.A.K.); pascal.vanvelthem@uclouvain.be (P.V.V.); wael.ballout@uclouvain.be (W.B.); bernard.nysten@uclouvain.be (B.N.); christian.bailly@uclouvain.be (C.B.); 2 Institute of Mechanics, Materials and Civil Engineering, UCLouvain, 1348 Louvain-la-Neuve, Belgium; nathan.klavzer@uclouvain.be; 3 Macromolecular Chemistry Unit, Applied Chemistry Laboratory, Faculty of Science, University of Yaoundé I, Yaoundé P.O. Box 812, Cameroon; mndikontar@yahoo.com

**Keywords:** PET, PLLA, blend, ecoselection

## Abstract

The structure and properties of blends of a novel polyethylene terephthalate copolymer (COPET) obtained by chemical recycling of commercial PET with high-molar-mass poly-L-lactide (PLLA) are investigated and compared to corresponding composites with chopped flax fibres. The focus is on the morphology at nano- and micro-scales, on the thermal characteristics and on the mechanical behaviour. The blends are immiscible, as evidenced by virtually unchanged glass transition temperatures of the blend components compared to the neat polymers (49 °C for COPET and 63 °C for PLLA by DSC). At low PLLA content, the blends display a sea–island morphology with sub-micron to micron droplet sizes. As the composition approaches 50/50, the morphology transitions to a coarser co-continuous elongated structure. The blends and composites show strongly improved stiffness compared to COPET above its glass transition temperature, e.g., from melt behaviour at 60 °C for COPET alone to almost 600 MPa for the 50/50 blend and 500 MPa for the 20% flax composite of the 80/20 COPET/PLLA blend. The flax fibres increase the crystallisation rate of PLLA in blends with dispersed PLLA morphology. The evidence of cavitation on the fracture surfaces of blends shows that despite the immiscibility of the components, the interfacial adhesion between the phases is excellent. This is attributed to the presence of aliphatic ester spacers in COPET. The tensile strength of the 80/20 blend is around 50 MPa with a Young’s modulus of 2250 MPa. The corresponding 20% flax composite has similar tensile strength but a high Young’s modulus equal to 6400 MPa, which results from the individual dispersion and strong adhesion of the flax fibres and leads close to the maximum possible reinforcement of the composite, as demonstrated by tensile tests and nano-indentation. The Ashby approach to eco-selection relying on the embodied energy (EE) further clarifies the eco-friendliness of the blends and their composites, which are even better positioned than PLLA in a stiffness versus EE chart.

## 1. Introduction

The escalating environmental pollution caused by uncontrolled dumping of plastic artefacts at their end of life, especially in the case of single-use items, has by now triggered numerous initiatives at national and supra-national levels to solve the problem. The European Union announced a target to increase recycling of plastics by at least 50% by 2030 [1].

Next to recycling industrial synthetic polymers, the development of bio-based and bio-degradable plastics such as poly(3-hydroxybutyrate) (PHB), poly(ethylene furanoate) (PEF), poly(caprolactone) (PCL), poly(butylene succinate) (PBS) and poly(lactic acid) (PLA) is receiving a lot of attention in academia and industry [2,3]. The recycling of industrial plastics and the development of bio-based polymers are both valid and complementary routes to mitigate the negative environmental impact of plastic waste and to move towards a circular organic materials economy. The recycling of existing synthetic plastics has a much higher short-term potential impact but lower long-term benefits towards a circular economy than entirely bio-based systems (polymer matrices as well as additives and reinforcing agents). In this study, an approach combining both avenues, based on blends and their flax fibre composites, made of poly L-lactide (PLLA) and a novel copolymer based on recycled poly(ethylene terephthalate) (PET) recently reported by Kuete et al. [4], was developed.

PET is one of the most widely consumed but also one of the most recycled plastics, at least in Europe [5]. PET’s main applications are single-use bottles for mineral water and carbonated soft drinks [6,7]. Industrially produced bottle-grade PET, with the highest molar mass, is already mechanically recycled at industrial scale to recover lower molar mass fibre-grade material for textile applications in a classical “downcycle” approach [8]. Chemical recycling of PET, although not yet advanced to industrial significance, has developed as well transformed post-consumer PET either back into PET repeating units or, more attractively, into chemical building blocks for “upcycled” high-value polymer products such as epoxy resins, alkyl resins, polyurethane resins, vinyl esters and polyester resins [9,10].

PLA is one of the most promising biopolymers that could, in the long run, replace synthetic polymers such as PET and polystyrene (PS) in food packaging [11]. PLA is already used to produce films, bottles, sheets and foams [12,13]. However, food applications of PLA are so far restricted by its poor oxygen barrier property and its very low toughness at room temperature [14,15]. The issue of low toughness of PLA is due in part to the presence of large spherulites, indicative of low nucleation density and resulting in slow crystallisation kinetics. Heterogeneous nucleation is the most common way to mitigate these issues. Generally, mineral fillers, e.g., talc particles, have been used as nucleating agents of PLA [16]. Nutenki et al. recently showed that flax fibres are also remarkable nucleating agents of PLLA, being able to induce a trans-crystalline layer of PLLA on their surface, as well as being effective reinforcing fillers due to their strong adhesion onto the polymer matrix [17].

**Figure 1:**
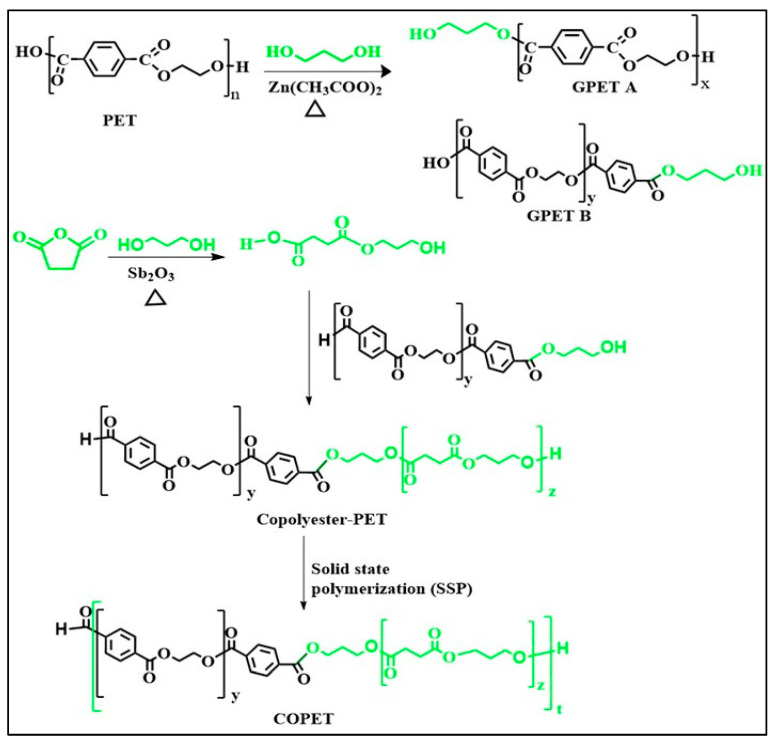
Reaction scheme of COPET synthesis (reproduced from Kuete et al. [4]).

Blending polymers with complementary properties is a widely used and low-cost strategy to optimize the performance of polymer materials by rebalancing the weakness of one component with the strength of the other [18]. The toughness and good barrier performance of PET clearly complement the low corresponding properties of PLA, while the crystallinity of PLLA can boost the thermal properties of the blends compared to amorphous PET alone. However, the natural tendency of polymers is to be highly incompatible with one another, because of the peculiar thermodynamic behaviour of dissimilar macromolecules when mixed, leading to ubiquitous incompatibility [19,20]. Hence, the expected benefits of polymer blends are not often achieved. Amorphous PLA has already been used as a nucleating agent of PET, but the compatibility between PET and PLA is poor even at low PLA content [3]. The two polymers are completely immiscible, and the resulting phases show poor interfacial adhesion, which is detrimental to the mechanical properties as the interfaces behave as internal defects [8]. Moreover, mixing PET and PLA in the melt by extrusion is tricky because the melting temperature of PET (around 260 °C) is close to the thermal stability limit of PLA, leading to PLA degradation during extrusion. Hence, it is desirable to lower the melt processing temperature of the PET component of the blend and concurrently improve its compatibility with PLA. This can be contemplated by introducing a moderate fraction of aliphatic ester in PET by copolymerisation. Such modifications have recently been performed by Kuete et al. [4] by incorporating aliphatic ester chain extenders in recycled PET chains, yielding a PET copolymer (COPET) from a partially glycolyzed starting PET with the incorporation of aliphatic moieties, the reactants being bio-based. To restore the mechanical properties, these reaction steps in the melt were followed by stepwise chain rebuilding in the solid state. The copolymers obtained were still able to crystallise, but very slowly. The glass transition temperature was moderately reduced to 48 °C, and the processing temperature was lowered to 180 °C, hence solving the risk of PLA degradation during melt processing [4]. The reaction scheme of the copolymer synthesis is summarized in Figure 1. The increased aliphatic ester content of the COPET significantly improved its compatibility with PLA as will be shown in the Section 3.

The objective of this work is twofold: (i) first to investigate the microstructure and major properties of the novel COPET-PLLA blends and their composites with flax fibres which have not been described before and (ii) second to substantiate the sustainability benefits of the blends and of their composites by following the materials selection method proposed by M. F. Ashby [21], which is based on a material index combining stiffness and embodied energy.

This work builds upon the previous contributions of Nutenki et al. [17] and Kuete et al. [4] who, respectively, studied PLLA-flax composites and COPET synthesis by chemical recycling of commercial PET followed by characterisation of its composites with banana fibres.

## 2. Materials and Methods

### 2.1. Materials

PLLA grade 2500 HP was obtained from Nature Works LLC in pellet form [22]. The intrinsic viscosity (*IV*) is 1.2 dL/g in a CHCl_3_–HFIP mixture according to the method described in Kuete et al. [4] for PET copolymers.

Unidirectional (UD) flax fibre mats (AmpliTex UD type 5057) were obtained from Bcomp (Fribourg, Switzerland). The mats were finely chopped in a Pulverisette mill 14 (Fritsh, Germany) to obtain fibres with 0.4–0.5 mm length.

The PET copolymer (COPET) used in this work was produced according to the combined melt and solid-state synthetic procedure described by Kuete et al. [4], named COPET11 in that paper. Accordingly, the copolymer had approximately 20 unperturbed PET units on average between the aliphatic spacers. The *IV* in the CHCl_3_–HFIP mixture mentioned above was 0.35 dL/g, much lower than that of PLLA 2500 HP, corresponding to a PET-equivalent M_v_ of 29,000 g/mol. The glass transition temperature measured by DSC was 49 °C.

### 2.2. Blend and Composite Processing

COPET and PLLA were dried overnight at 60 °C under vacuum to remove moisture and prevent polymer degradation by hydrolysis following the recommendation of the supplier (Nature works technical data sheet). Ground fibres (100 < diameter < 300 µm) were dried at 105 °C under vacuum for 12 h. Blends of COPET containing 10, 20 and 50% *w*/*w* PLA were first compounded in twin-screw micro compounder Xplore MC 15 (DSM, Sittard, The Netherland) operated in batch mode with a recirculation channel allowing a closed-circuit travel of the molten compound for the chosen period before discharge. The conical twin screws of the extruder are positioned vertically, with the plane defined by the screw axes are oriented front to rear when the operator is facing the machine. Three independent temperature regulation zones are provided from top to bottom for both the front and the rear of the machine. A seventh zone is reserved for the die. The top zone is for feed, the middle zone for compression, and the bottom zone for plasticization. The chosen temperatures were 180 °C–190 °C–200 °C from top to bottom and 200 °C for the die. The recirculation channel allows closed-circuit travel of the molten compound for the chosen period before discharge. The chosen temperatures were 180 °C–190 °C–200 °C from top to bottom and 200 °C for the die. The screw speed was 100 rpm, and the residence time in the extruder was about 2 min. The extruded blends were pelletised in a Varicut 11 mm pelletiser (ThermoFisher, Karlsruhe, Germany) and dried at 50 °C under vacuum for 24 h before injection moulding to produce dog-bone test pieces (ISO 527-2-5A) in a Thermo-Scientific HAAKE MiniJet Pro (ThermoFisher, Karlsruhe, Germany). The cylinder temperature was set at 190 °C, the mould temperature at 50 °C, the pressure at 600 bars and the post-pressure at 500 bars, all maintained for 30 s. The processing temperature is slightly lower than the recommendations of the supplier (205 °C) to avoid degradation during the process.

For the composites, PLLA was first dissolved in chloroform, and various amounts of chopped flax fibres (5, 10 and 20% *w*/*w*) were added to the solution; next, the chloroform was evaporated. The rest of the procedure was identical to the one described above for the blends.

### 2.3. Characterisation Techniques

#### 2.3.1. Differential Scanning Calorimetry (DSC)

A differential scanning calorimeter DSC1 (Mettler Toledo, Greifensee, Switzerland) was used to determine the glass transition temperature (*T_g_)*, the melting temperature (*T_m_*), the melting enthalpy (Δ*H_m_*) the crystallinity (*X_i_*) and the crystallisation temperature (*T_c_*) or isothermal kinetics of the samples. DSC analyses were carried out under nitrogen gas with sample masses of about 8 mg. The investigated temperature range of the first heating was between 25 and 220 °C at a rate of 10 K/min, followed by a cooling from 220 to 25 °C at −20 K/min to avoid cold crystallisation, and a second heating was performed from 25 °C to 220 °C at a rate of 10 K/min. The procedure for the isothermal kinetics study consisted of an initial cooling step as above, followed by heating from 25 to 100–130 °C at 20 K/min and maintaining this temperature for 2 to 15 min before cooling to 25 °C at −20 K/min and a final heating from 25 °C to 220 °C at 10 K/min. Glass transition temperatures (*T_g_*), enthalpy of cold crystallisation ($H_{cc}$), melting enthalpy ($H_{m}$) and melting temperature (*T_m_*) were determined from first and second heating scans [23]. Degrees of crystallinity ($X_{i}$) of PLLA in the blends were calculated from the following equation [24]:

$$\chi_{PLA}=\frac{{∆H}_{m}-{∆H}_{cc}}{\omega_{i}\times {∆H}_{mPLA}^{{}^{\circ}}}$$

where ${∆H}_{m}^{0}$ = 93.7 J/g is the melting enthalpy of neat PLA at 100% crystallinity [17], and $\omega_{i}$ is the weight fraction of PLA.

#### 2.3.2. Polarized Light Optical Microscopy

An Ax70 microscope (Olympus, Tokyo, Japan) was used in polarized light mode to observe COPET, PLLA, blends and composites. An FP90 hot stage (Mettler Toledo, Greifensee, Switzerland) was used to impart the desired thermal history to the sample. Samples were placed between two glass slides, heated from 25 to 220 °C at 20 °C/min and then kept at this temperature for 1 min to allow complete melting, before quickly cooling down to 50 °C to prevent crystallisation. Next, the samples were heated from 25 °C to 100–130 °C at 20 K/min and kept at this temperature for 2 to 15 min before cooling to 25 °C at −20 K/min. Finally, the samples were heated from 25 °C to 220 °C at 10 K/min to obtain the crystallisation enthalpy after annealing.

#### 2.3.3. Scanning Electron Microscopy (SEM)

SEM analyses were performed on fracture surfaces of tensile test specimens at room temperature or under cryogenic conditions with the help of a JEOL 7600F microscope (JEOL Ltd., Tokyo, Japan) operated at 1.5 kV. The fracture surfaces were coated with platinum by sputtering to avoid electrical charging.

#### 2.3.4. Atomic Force Microscopy (AFM)

AFM analyses of the COPET-PLLA blends were performed on trimmed surfaces with a Dimension Icon system (Bruker Corp., Billerica, MA, USA) in PeakForce Tapping mode with Quantitative Nanomechanical Mapping (PFT-QNM). A factory-calibrated probe, RTESPA-300–30 (Bruker Corp., Billerica, MA, USA), with a spring constant of 48.87 N m^−1^ and a tip apex radius of 34 nm was used. AFM images (256 × 256 pixels) with dimensions ranging between 3 × 3 and 30 × 30 µm^2^ were acquired. The PeakForce frequency and amplitude were 2 kHz and 30 nm, respectively. The PeakForce set point was set to 75 nN, corresponding to an indentation depth between 2 and 3 nm. Under these conditions, the contact radius remained smaller than 10 nm [25].

#### 2.3.5. Dynamic Mechanical Analyses (DMA)

The dynamic mechanical properties of the polymer blends and composites were analysed in tensile mode on a DMA/SDTA861 testing machine from Mettler Toledo (Greifensee, Switzerland), equipped with a 40 N load cell. The samples were obtained by cutting dog-bone mouldings to average size (9 × 4 × 2.5 mm^3^) and annealed for 10 min at 110 °C before analysis. The heating scans were performed at a constant frequency of 1 Hz at 3 K/min scan rate from 10 to 150 °C, maximum force of 2 N and maximum displacement of 1 cm. Measurements were performed on at least three samples for reproducibility.

#### 2.3.6. Nano-Indentation

Nano-indentation tests were performed using a Nano-indenter G200 (KLA Tencor, Milpitas, CA, USA) with a dynamic contact modulus (DCM V2) head giving high precision at low loads and displacements. The maximum force on the device was set to 45 mN with a resolution of 1 nN. The diameter of the Berkovick tip was 1000 nm; the depth limit was 500 nm. Before analysis, the samples were embedded into epoxy resin and then polished on a semi-automatic MultiPrep Precision Polishing System (Allied High Tech Products, Inc., Cerritos, CA, USA). Different discs with successive grits (MD-Piano from Struers Inc., Westlake, OH, USA) were used for polishing, and 0.3 µm alumina suspension (AP-D Suspension 0.3 µm, Struers Inc., Ballerup, Denmark) was used for the finishing.

#### 2.3.7. Tensile Tests

Tensile tests were carried out on as-moulded neat polymer, 80/20 COPET-PLLA blends and their composites after annealing. The samples were tested at a relative humidity of 50% at room temperature on a Zwick Roel test machine at a crosshead speed of 5 mm/min. These tests were performed on 5 specimens for each batch.

## 3. Results and Discussion

All characterisation and testing experiments discussed in this section were carried out on (pieces taken from) dog-bone mouldings obtained by injection moulding of as-received samples in the case of neat polymers or melt-compounded blends and composites, as detailed in the experimental section. Some blend and composite mouldings were first annealed before testing to increase the crystallinity of the PLLA fraction.

### 3.1. Thermal Properties by DSC

**Figure 2:**
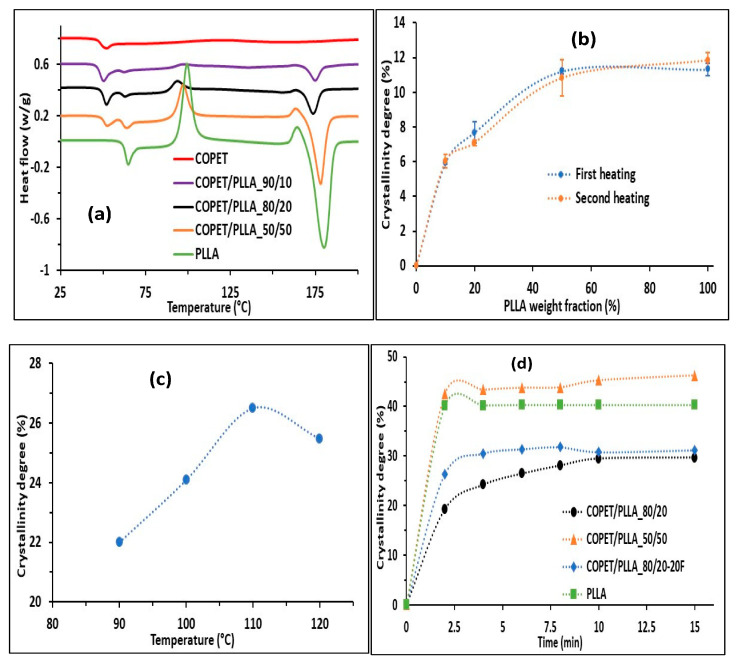
Thermograms of the COPET-PLLA blends. First heating scans (**a**), PLLA crystalline fraction vs. PLLA fraction in blend (**b**), PLLA crystalline fraction vs. annealing temperature (**c**) and isothermal crystallisation kinetics at 110 °C (**d**).

DSC runs of as-moulded and annealed COPET-PLLA blends were compared to neat reference polymers to clarify the influence of blending on the thermal transitions and the PLLA crystallisation kinetics. The results of first heating runs are presented in Figure 2a below.

Figure 2a shows the glass transition temperatures of neat COPET and PLLA at 49 °C and 63 °C, respectively. On the other hand, the blends display two glass transitions that are very slightly shifted compared the neat polymers, at 51 °C and 62 °C, respectively. The blends are clearly biphasic. The question of a slight COPET-PLLA inter-miscibility cannot be unambiguously decided from DSC alone because the enthalpic relaxation endotherms observed just beyond the delta *C*_p_ steps obscure the exact locations of the *T_g_*. The issue is further discussed in Section 3.5., which is devoted to DMA analyses, showing that the COPET-PLLA inter-miscibility is negligible. The same result has been obtained in a related system with commercial PET and amorphous PLA [8].

Under the thermal programme rates of the experiments, the COPET remained amorphous, but PLLA was able to crystallise. The cold crystallisation temperature (*T_cc_*), the enthalpy of cold crystallisation (${∆H}_{cc}$), the melting temperature (*T*_m_) and the melting enthalpy (${∆H}_{m})$, corrected for PLLA content, all increased with the PLLA fraction in the mixture. Figure 2b shows that the PLLA crystalline fraction in the blends after moulding (i.e., first DSC runs) increased from 6% to 11% when the PLLA content of the blend increased from 10 to 50% and stayed constant at a higher PLLA fraction. This result is consistent with the nucleation-limited crystallisation of a dispersed PLLA phase in a COPET matrix below 50% PLLA, shifting to co-continuity or PLLA matrix at higher PLLA. This will be fully confirmed by the microscopy study presented below (Section 3.2). The crystallisation kinetics during the second heating (first run samples quenched after melting) are undistinguishable from the first runs.

To further increase the PLLA crystalline fraction, annealing programmes were tested on as-moulded 80/20 COPET-PLLA blends. The annealing temperature was varied between 90 and 120 °C to favour the α-crystal form of PLLA (Figure 2c). Maximum crystallinity was achieved at 110 °C. This temperature is 10 °C lower than the corresponding maximum crystallinity temperature obtained by Nutenki et al. [17] on pure PLLA. The most logical explanation for this difference is a stronger nucleation vs. growth limitation in the blends with dispersed PLLA as opposed to a continuous PLLA matrix.

Finally, the crystallisation kinetics and final crystallinity at 110 °C of neat PLLA, COPET-PLLA 80/20 and 50/50 were measured and compared to the COPET-PLLA 80/20 composition reinforced by 20% flax fibres (Figure 2d). The figure demonstrates the nucleation ability of the flax fibres as well as the favourable influence of a continuous or co-continuous PLLA phase (faster kinetics and higher final crystalline fraction).

### 3.2. Result of Polarized Light Optical Microscopy

**Figure 3:**
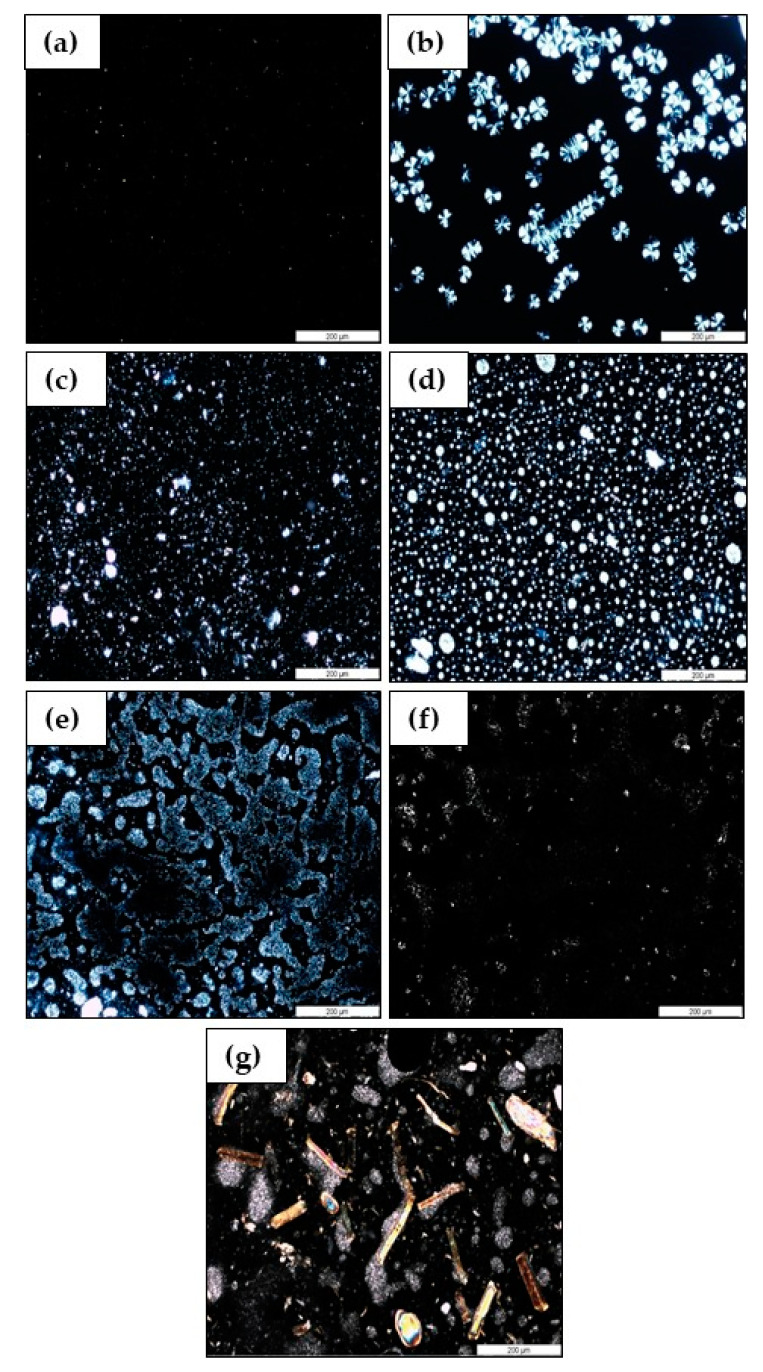
Optical microscopy of COPET (**a**), PLA (**b**), COPET/PLLA_90/10 (**c**), COPET/PLLA_80/20 (**d**), COPET/PLLA_50/50 (**e**), COPET/PLLA_80/20 after micro-filtration (**f**) and composite COPET/PLA_80/20-5F (**g**).

Optical microscopy observations under polarized light were carried out after annealing at 110 °C on COPET, PLA and COPET-PLLA blends (90/10, 80/20 and 50/50) and the 80/20 composite with 5% flax *w*/*w*. The results are in Figure 3.

Figure 3a shows that COPET remained amorphous after annealing. The tiny white dots are undoubtedly crystalline catalyst particles since the catalyst was not removed. Figure 3b of neat PLLA shows a partially crystallised matrix after annealing. The identical size of all spherulites indicates that nucleation was heterogenous and instantaneous, rather than sporadic. Figure 3c,d shows that COPET-PLLA-90/10 and 80/20 blends were characterised by crystallised PLLA droplets dispersed in a COPET amorphous matrix. The size of the dispersed droplets increased when the PLLA fraction increased. This is a well-known coalescence effect in polymer blends with a sea–island morphology at increasing concentration of the dispersed phase. The same results were observed in other PLA blends (PLA/ polycaprolactone) [26] and PLA/polybutylene adipate terephthalate [27]. At 50% PLLA (Figure 3e), the morphology of the blend became co-continuous, confirming the tentative conclusion drawn from DSC (Figure 2b,d). Well-dispersed crystalline PLLA droplets dispersed in a COPET continuous matrix should already provide some improvements in the thermomechanical performance vs. amorphous COPET alone, but a co-continuous morphology should further be advantageous [27]. The same conclusions will also be obvious from DMA analyses presented in Section 3.5. The effect of the residual COPET polymerisation catalyst was studied for the 80/20 COPET-PLLA blend by comparing unfiltered (Figure 3d) and 0.2 µm micro-filtered samples (Figure 3f). The catalysts used in the synthesis of COPET by PET chemical recycling were 0.1% wt zinc acetate (Zn(CH3COO)2) and 0.1% wt antimony oxide (Sb2O3) [4]. Lower residual concentrations must be present in COPET after the polymer workup process and further diluted in COPET-PLLA blends in proportion to the COPET concentration and to an unknown partition coefficient. Metallic oxides and zinc metal salts have already been reported as nucleating agents of PLLA. Among those, the most effective ones are titanium oxide (TiO_2_), zinc oxide (ZnO) [28], zinc phenyl phosphonate, zinc citrate, zinc phenyl malonate and zinc salts of amino acids [29,30]. To our knowledge, antimony oxide and zinc acetate are not known as a very effective nucleating agent of PLLA, but their nucleation ability should not come as a complete surprise from the references mentioned above. After filtration, the amount of crystalline PLLA droplets was much lower than before filtration. This confirms that the residual catalyst (Sb2O3 and Zn(CH3COO)2) in COPET acts as an effective nucleating agent of PLLA.

The peculiar morphology after annealing of the 80/20 COPET-PLLA composite with 5% flax fibres is illustrated in Figure 3g. Crystalline PLLA domains remained mostly attached to the fibres. To understand this feature, it is necessary to go back to the procedure for the preparation of the composite. As explained in the Section 2 the flax fibres were pre-coated by PLLA from solution before compounding with COPET in order to maximize the favourable surface interactions between the two, as previously observed by Nutenki et al. [17]. Because the *IV* of the PLLA is much higher than that of the COPET (1.2 dL/g vs. 0.35 dL/g), the corresponding melt viscosities must be quite different since the molar mass scaling is much higher for the melt viscosity. A naïve but reasonable estimate (0.8 vs. 3.4 molar mass scaling powers for *IV* vs. melt viscosity and comparable proportionality factors for the two polymers) suggests a factor above 20 for the melt viscosity ratio. Hence, the PLLA coating the flax fibres did not become efficiently dispersed in the matrix during compounding because of the viscosity mismatch (highly viscous phase dispersed in a more fluid matrix). Moreover, there is no evidence of a transcrystalline phase on the flax fibres because the annealing conditions of the composites favoured nucleation in the bulk.

### 3.3. Result of Scanning Electron Microscopy (SEM)

**Figure 4:**
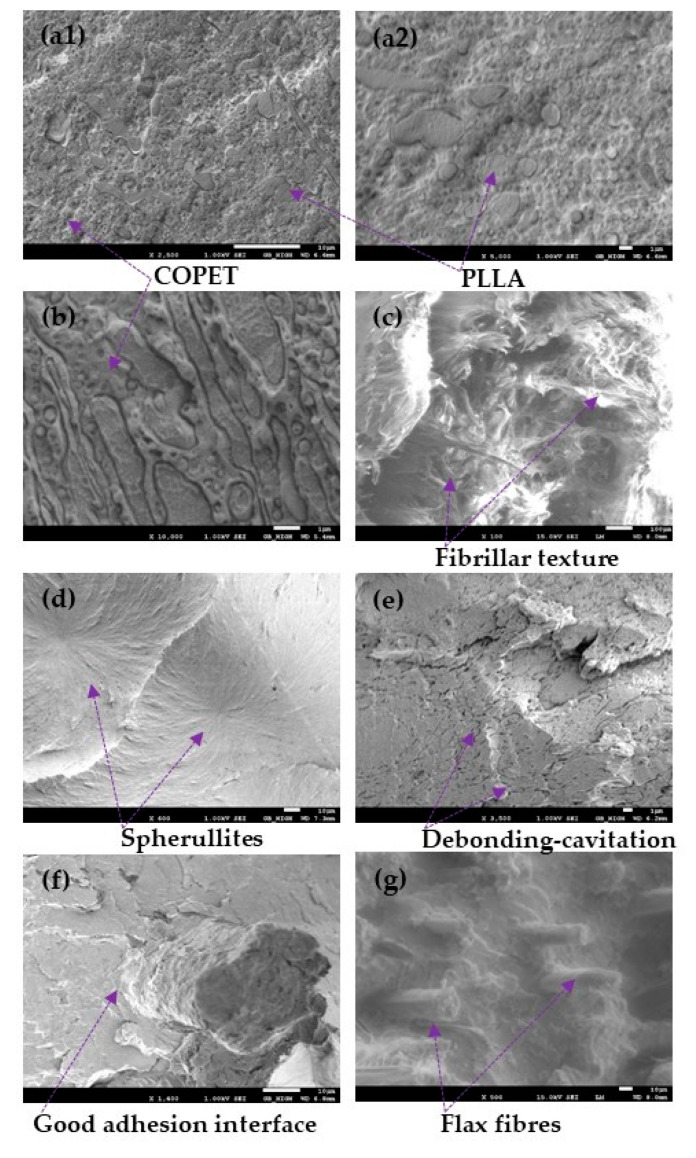
SEM micrographs of fracture surfaces obtained at cryogenic temperature: COPET/PLLA_80/20 (**a1**,**a2**) and COPET/PLLA_50/50 (**b**); RT fracture surfaces after tensile tests: COPET (**c**), PLLA (**d**), COPET/PLLA_80/20 (**e**) and composites COPET/PLLA_80/20-5F (**f**,**g**).

SEM micrographs were taken of COPET-PLLA 80/20 and 50/50 blends after annealing and fracture at cryogenic temperature as well as on RT fracture surfaces of annealed dog-bones after tensile tests of COPET, PLA and COPET-PLLA_80/20 blends and the corresponding composite with 5% flax fibres. The two kinds of fracture surfaces are presented in Figure 4.

Figure 4a1,a2 shows mostly micron and sub-micron dispersed PLLA domains in the COPET-PLLA 80/20 blend. The cracking path at cryogenic temperature has propagated through the dispersed domains instead of following the interfaces, which confirms the high interfacial adhesion. Moreover, the high hydrostatic stresses produced alongside growing cracks favoured the formation of cavitation-induced interfacial voids [27]. Figure 4b highlights the co-continuous phase morphology when the PLLA fraction is around 50%. This agrees with the optical microscopy results. Moreover, the morphology is highly elongated, which is due to the high viscosity contrast between the two phases and should favour barrier properties.

Figure 4c,d shows neat COPET fractures at room temperature in a very ductile manner with a fibrillated texture. Figure 4d of neat PLLA shows a highly brittle fracture exposing large spherulite clefts through the centre.

Figure 4e reveals that the debonding–cavitation mechanism relaxes hydrostatic stresses and hence mitigates the risk of crazing while favouring the development of plastic shear band, which is also dominant at room temperature as observed for the COPET/PLA_80/20 blend. A classic example in the literature of a similar mechanism is the poly(butylene terephthalate)/polycarbonate blend [31]. However, Figure 4e does not reveal extensive plastic deformation before fracture. This observation is consistent with the stress–strain curves shown in Section 3.7. Figure 4f,g shows that after annealing, the interface between the fibres and the matrix in the composites (COPET80/PLA20-5F) was quite strong. Similar results have been obtained for composites made of PLLA with flax fibres [17] and COPET with banana fibres [4].

### 3.4. Result of Atomic Force Microscopy (AFM)

**Figure 5:**
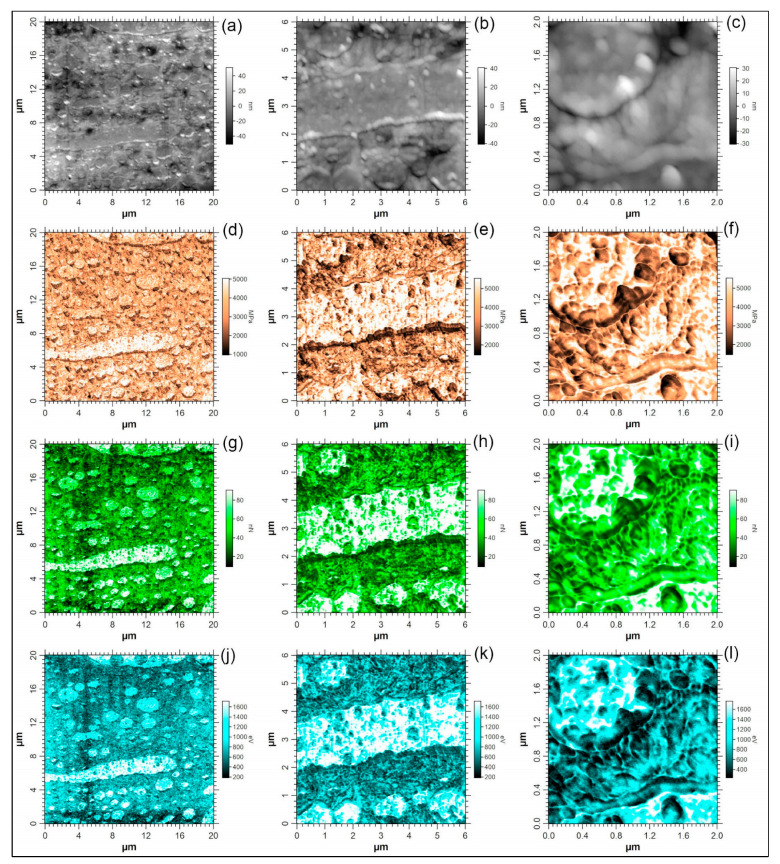
AFM images obtained in PFT-QNM mode on the COPET/PLLA_80/20 blend on the same region at different magnifications. Topography images (**a**–**c**), DMT modulus images (**d**–**f**), adhesion force images (**g**–**i**) and energy dissipation images (**j**–**l**).

Atomic force microscopy analyses were performed on a smooth dressed surface of the COPET-PLLA 80/20 blend to understand the dispersion of PLLA in COPET at the micro/nanoscale. PFT-QNM was used to simultaneously generate topographic, stiffness (DMT modulus), adhesion force and energy dissipation images. The results are presented in Figure 5a. Ten force–displacement curves for the matrix assumed to be COPET and ten curves of the droplets assumed to be PLLA were selected randomly. The PLLA and COPET curves were averaged over each of the two phases, as shown in Figure 5b.

Figure 5 clearly shows that there are inclusions (droplets) of varying sizes with higher adhesion force and energy dissipation than the continuous phase. The fact that the DMT modulus on these inclusions was slightly higher than on the continuous matrix suggests that these are PLLA-rich inclusions since the macroscopic modulus of semi-crystalline PLLA was larger than that of COPET (Section 3.5). The average values of the adhesion force, *F*_adh_, and of the DMT modulus, *E*_DMT_, were determined both on the continuous phase and on the inclusions. On the continuous phase, the following values were obtained: *F*_adh_ ≈ 22.5 ± 6.3 nN and *E*_DMT_ ≈ 1480 ± 640 MPa; on the inclusions, *F*_adh_ ≈ 82.6 ± 6.5 nN and *E*_DMT_ = 3380 ± 360 MPa. The values of the modulus are compatible with those obtained in tensile and DMA tests, around 2000 MPa for COPET and around 3000 MPa for PLLA.

No voids were observed at the interphase between PLLA and COPET, indicating a good interphase compatibility. This contrasts with the observation reported in the literature on PET-amorphous PLA [8,32], which showed a partly delaminated interface. The improved compatibility is likely due to the additional aliphatic ester groups present in COPET, leading to a better matching of the solubility parameters.

### 3.5. Thermomechanical Properties (DMA Analyses)

**Figure 6:**
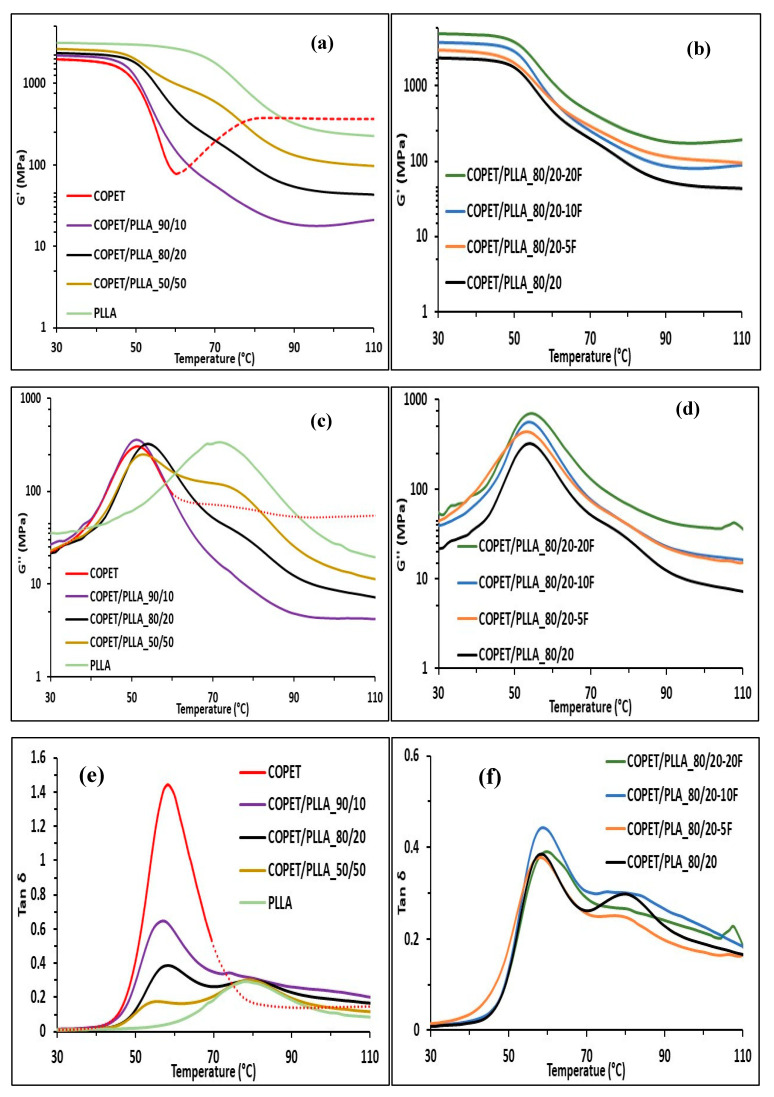
Storage moduli (**a**,**b**), loss moduli (**c**,**d**) and tan δ (**e**,**f**) of COPET-PLLA blends and of COPET/PLLA_80/20-xF composites.

DMA analyses were performed on specimens cut from tensile dog-bone specimens after annealing, as detailed in the Section 2. The results are presented in Figure 6.

Figure 6a shows that the storage modulus above the glass transition temperature clearly increases with increasing PLLA concentration because the annealed PLLA is highly crystalline and acts as a reinforcing filler up to its melting point. The effect is very pronounced in the 50/50 blend, owing to its co-continuous morphology (Figure 4b). For instance, at 60 °C, neat COPET behaves as a melt, but the 50/50 blend has a storage modulus of almost 600 MPa (Figure 6a). This validates the interest in the blending approach used in this study. The storage modulus scan of neat COPET (Figure 6a) indicates that above *T_g_*, the polymer crystallises slowly during the DMA analyses. However, the apparent modulus increase is not reliable because the sample was deformed in the instrument during the scan. Some of the blends and composites showed a similar but much less pronounced trend and were considered reliable.

Figure 6b shows that the storage modulus below *T_g_* of the COPET-PLLA 80/20 system strongly increases with increasing flax content from 2300 MPa for the unreinforced blend to 5100 MPa for the 20% flax composite. Above *T_g_*, the modulus drastically decreases as expected but the benefit of flax fibres was clearly visible. For instance, the 20% flax composite of the 80/20 COPET/PLLA blend has a storage modulus of 500 MPa at 60 °C (Figure 6b), whereas neat COPET is a melt. This validates the reinforcement strategy developed in this work. The excellent dispersion of the fibres in the polymer (Figure 4g,f) played a favourable role in this respect. Figure 6c–f shows that the glass transition temperatures of neat PLLA and COPET measured from the damping peaks are, respectively, 78 °C and 58 °C. The loss modulus (G”) and damping peak temperatures (tan δ) of the blends and their composites do not show a systematic trend that would suggest even a slight inter-miscibility. The blends are therefore fully immiscible, but both SEM and AFM results discussed above indicate that the interfacial adhesion between COPET and PLLA is good. A word of caution is warranted here. The DMA test pieces were annealed before the measurements, and it is possible that this procedure erased whatever minute inter-miscibility there was after moulding. However, DSC on unannealed samples (Figure 2) shows a maximum shift of 2–3 °C for the blends COPET/PLLA compared to neat COPET. Such small changes are at the limit of DSC discrimination capability and, even if real, only have a very minor effect on the thermo-mechanical properties.

Finally, the independence of measured *T_g_* on fibre content suggests that chain mobility reduction by the matrix–fibre interface was insignificant due to the micro- rather than nano-diameter of the fibres (no nanocomposite chain confinement effect).

### 3.6. Result of Nano-Indentation Tests

**Figure 7:**
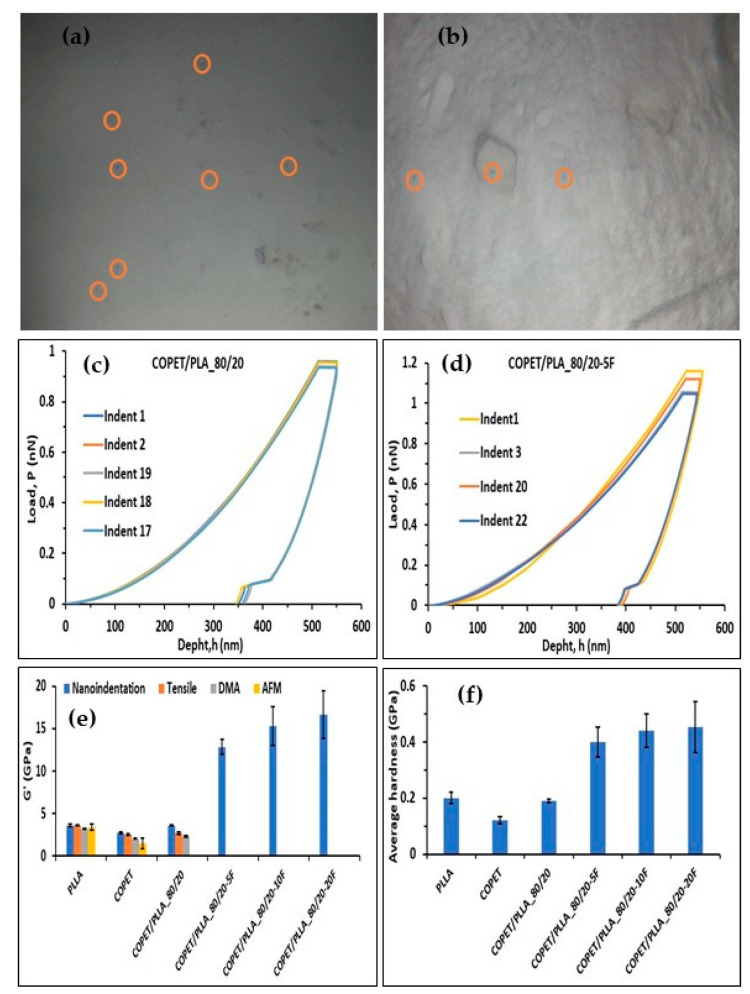
Micrographs of nano-indentation imprint of matrix (**a**) and fibres (**b**), nano-indentation penetration of matrix (**c**) and fibres (**d**), Young’s modulus (**e**) and hardness (**f**) at indentation loads of 500 nN at a loading rate of 0.05 mN s^−1^.

Nano-indentation tests were performed on polished surfaces of annealed moulds of the neat polymers and composites of the COPET-PLLA 80/20 blend. The surfaces were indented at room temperature (25 °C) at 0.05 mN/s constant loading rate by applying a 10 mN maximum load to the depth in order to identify the moduli of the COPET, PLA and fibres (Figure 7a,b). Typical load–penetration curves are shown in Figure 7c,d. The extracted Young’s modulus and nano-hardness are shown in Figure 7e,f.

Figure 7e,f shows that the Young’s modulus and nano-hardness of PLLA were higher than those of COPET and the COPET-PLLA-80/20 blend. This was due to the crystallinity of PLLA as opposed to the amorphous character of COPET. The elastic modulus (*E*’) values obtained by nano-indentation are different from those obtained by tensile testing, DMA and AFM due to the solicitation scale and to the geometry of the nano-indenter but are reasonably consistent with the macroscopic values extracted from DMA and from tensile tests. The indentation performed on single fibres surrounded by the blend COPET/PLA-80/20 gave a modulus of the fibres around 17 ± 2 GPa. This value is consistent with the literature [33]. Transverse and longitudinal properties of the fibres were different due to their anisotropy nature. The nano-indentation modulus was lower than the tensile modulus of the fibres. One reason is the significant anisotropy of the fibres and the fact that the loading condition under the indentation tip was multiaxial, not allowing the extraction of the “pure” modulus in the fibre axis direction. In tensile tests, microfibrils were pulled and oriented in the longitudinal direction, while in nano-indentation tests their orientation remained the same.

### 3.7. Result of the Tensile Tests

**Figure 8:**
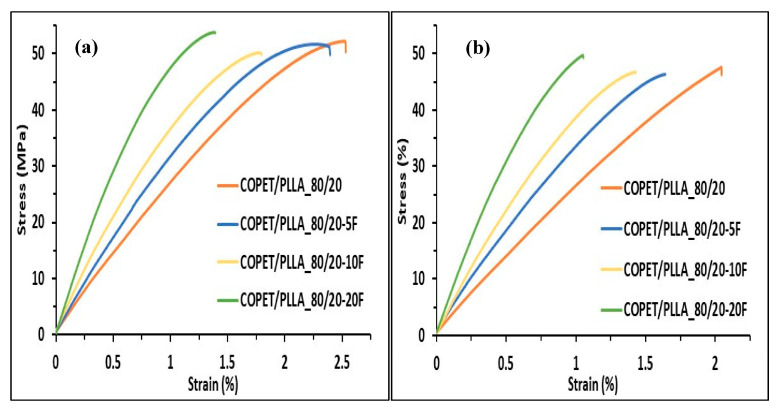
Tensile properties of COPET-PLLA_80/20 and flax fibre composites (COPET-PLLA_80/20-xF) at 25 °C before annealing (**a**) and after annealing (**b**).

Tensile tests were performed on as-moulded and annealed dog-bone specimens to understand the influence of the fibres’ fraction and annealing on the macroscopic mechanical properties. The results are presented in Figure 8.

Figure 8 shows that the tensile strength of the 80/20 COPET-PLLA blend and of the composites was similar (around 50 MPa), clearly pointing to internal defects, either in the bulk phase or interfacial, limiting the resistance to failure. This value of tensile strength is similar to related systems. The only published results on the COPET copolymers studied in the present article are from our own team (Kuete et al., 2022, reference [4]). The tensile strength of the neat copolymer is about 45 MPa, 10% less than the PET reference at 50 MPa. These values are typical of amorphous semi-aromatic polymers below Tg. Composites (with chopped banana fibres) in the same article have a tensile strength comprised between 40 and 50 MPa. R. Nutenki et al. (reference [17]) measured a tensile strength of 72 MPa before annealing and 67 MPa after annealing for the same PLLA as used here. The values for the PLLA composites with chopped flax fibres studied in that paper (60–67 MPa depending on composition) are in turn compatible with the results obtained earlier by Aliotta et al. [34] and the citations in that paper, i.e., between 50 and 70 MPa depending on the fibres’ concentration. The blends and composites studied in the present paper show compatible values of 45 to 55 MPa with these references. Zhang et al. [35] have further published the tensile strength of a related system PLLA/poly butylene succinate without annealing and shown that the tensile strength of the 20/80 blend is comprised between 50 and 53 MPa. The tensile strength results that we obtained in the present paper (50 MPa without annealing) are in the same range.

Overall, our results are hence in line with the literature, keeping in mind the differences between compositions and processing details across the articles.

**Figure 9:**
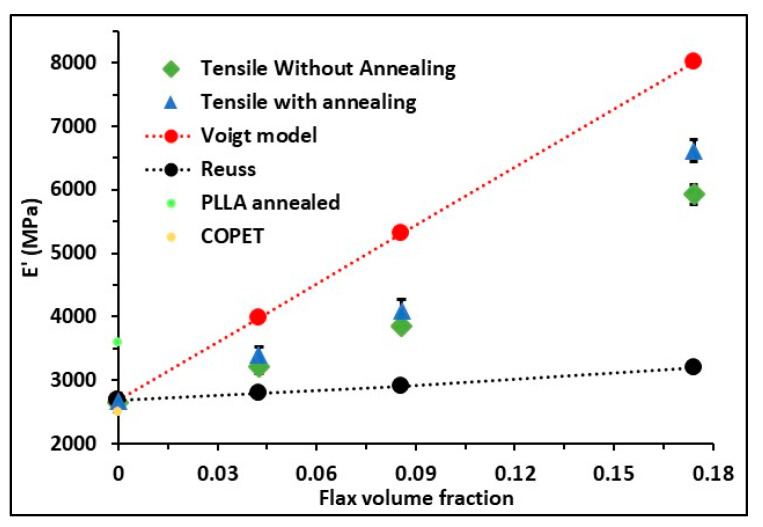
Tensile properties of COPET-PLLA 80/20 and composites (COPET-PLLA-80/20-xF) at 25 °C as a function of the fibre fraction: experimental Young’s modulus vs. fibre content, compared to Voigt and Reuss model bounds.

Based on the hardness measured by the nano-indentation equal to 190 MPa for the annealed COPET/PLLA80/20 (see Figure 7f), one expects a much higher yield strength in the order of 75 to 110 MPa (knowing that the conversion factor between hardness and yield strength typically varies between 1.75 and 2.5 for polymers [36,37]). Fracture occurs before overall plasticity takes place, even though some regions may locally yield in the composite at specific inter-fibre locations of stress concentrations [38]. Fracture is thus not the result of damage accumulation by (visco-)plastic deformation such as in more ductile polymer systems. Note finally that the fracture strain is reduced when the concentration of fibres increases as a result of a higher modulus. Figure 9 shows that the tensile modulus of the composites increased from 2.6 GPa to 6.4 GPa when the fibre fraction increased from 5 to 20%. The experimental Young’s modulus of the composites (before or after annealing) was comprised between the Reuss and Voigt theoretical bounds but closer to the Voigt upper bound. This comes from the fact that fibres are mostly oriented parallel to the length of the test specimens due to the melt flow in the mould during injection, leading to quasi iso-strain conditions. Annealing treatment of the composites contributes to an increase in the modulus by enhancing the PLLA crystallinity in the blend and in the composites.

### 3.8. Sustainability Analysis

The materials selection method proposed by M.F. Ashby [21] was used to construct a material index reflecting minimization of embodied energy under a beam stiffness constraint. The relevant material index *M* for an objective to minimize environmental impact is as follows [21]:

$$M=\frac{\rho H_{p}}{E^{1/2}}$$

where ρ is the density (kg/m^3^), *H_p_* is the embodied energy per mass units (MJ/kg) and *E* is the Young’s modulus. Embodied energy essentially represents the energy consumed to make one kilogram of material (including extraction, manufacture and transport), and it generally lies in the range of 50 to 250 MJ/kg for polymers. This value is reduced for recycled materials. Embodied energy of a recycled material (10–100 MJ/kg) is lower because the intrinsic energy of the first production is not included in this case, which is a valid assumption if many recycling steps are envisaged.

**Figure 10:**
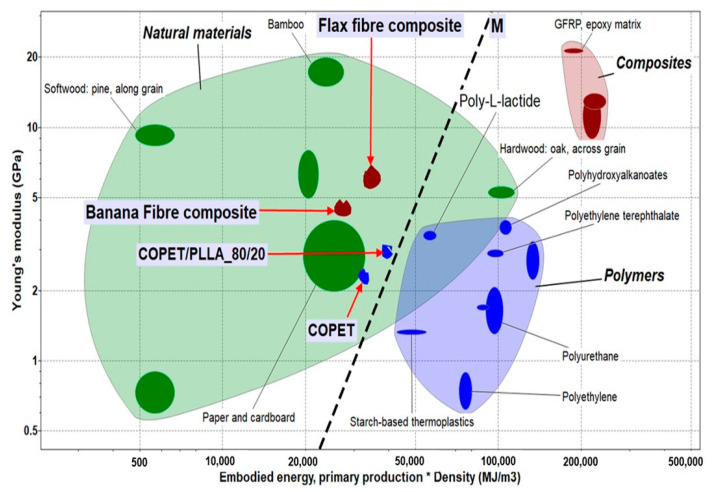
Selection chart for stiffness vs. embodied energy (after screening for irrelevant materials) including COPET, 80/20 COPET/PLLA blend and corresponding composite with 20% flax fibres.

The composites developed in this work were included in the relevant materials property map (Figure 10).

It is important to keep in mind that the sustainability assumption for COPET is based on recycled PET. The list of relevant materials in this chart includes COPET based on recycled PET, PLLA (not recycled), flax fibres and blends/composites of COPET, PLLA and flax fibres. The materials with iso performance by reference to the material index *M* reside on the straight lines parallel to the dashed line in Figure 10. The density-corrected embodied energy values extracted from the EDUPACK, 2022 software database [39] are as follows:

*Embodied energy* × *density PET* = *100,000 MJ/m^3^*

*Embodied energy* × *density COPET* = *34,710 MJ/m^3^*

*Embodied energy* × *density PLA (amorphous or crystalline)* = *56,896 MJ/m^3^*

*Embodied energy* × *density COPET/PLLA_80/20* = *39,719 MJ/m^3^*

*Embodied energy* × *density COPET/PLLA_80/20_20F* =*35,435 MJ/m^3^*

*Embodied energy* × *density Flax* = *15,400 MJ/m^3^*

The figure clarifies that the COPET-PLLA blends have (i) a lower embodied energy than PLLA alone but slightly higher than COPET, quantifying the benefit of including recycled polymer in the formulation, but (ii) a lower Young’s modulus. The flax composites of the blends are favourably positioned inside the “natural materials” domain with high stiffness and low embodied energy. In conclusion, as long as recycled polymers can be considered as genuine raw materials in new applications (which is debatable, but the discussion is beyond the scope of this work), their sustainability benefits are huge, even higher than for pure biomaterials such as PLLA. Moreover, the COPET-PLLA blends and their composites have higher temperature capability than the COPET equivalents (see Figure 6a,b). Hence, the blending strategy used in this work makes real sense.

## 4. Conclusions

In this study, the morphology from nano- to micro-scale and the (thermo-)mechanical properties and the sustainability benefits of non-reinforced as well as flax-reinforced blends of PLLA with a recently developed PET copolymer (COPET) were investigated.

COPET-PLLA blends were (almost) completely immiscible (maximum 2–3 °C change of *T_g_* in blends compared to neat components) but showed good interfacial adhesion, as demonstrated by cavitated fracture surfaces and micron–sub-micron dispersion of the dispersed PLLA phase (in 80/20 COPET/PLLA blends), resulting in ductile unreinforced and reinforced materials, as demonstrated by DSC, AFM and SEM, leading to some ductility of unannealed blends and composites. A reasonable explanation for fine morphology and good properties is the presence of aliphatic ester spacers in COPET, which improves compatibility with PLLA even in the absence of significant inter-miscibility.

The presence of PLLA remarkably increases the storage modulus of the blends above *T_g_* compared to COPET alone, e.g., from melt behaviour at 60 °C for COPET alone to a 500 MPa soft solid for the 50/50 blend at the same temperature.

Although crystallisable, COPET remained amorphous under usual melt-processing conditions, as opposed to PLLA. The crystallisation rate of PLLA in the blends with a dispersed PLLA phase is increased compared to the unreinforced blends demonstrating the nucleation ability of the fibres, e.g., primary crystallisation at 110 °C completed in 4 min for the 20% flax composite vs. 10 min for the same 80/20 blend composition. The residual COPET polymerisation catalyst also acts as a nucleating agent.

Below *T_g_*, the flax fibres have a remarkable reinforcing effect on the composites, e.g., 6400 MPa for the 80/20 COPET/PLLA blend with 20% flax fibres compared to 2300 MPa for the unreinforced blend.

The eco-selection approach of M.F Ashby was further used to validate the eco-friendly character of the blends. The embodied energy vs. stiffness chart shows that COPET-PLLA blends are even better positioned than PLA, provided that COPET is based on recycled PET and the latter can be considered as a true raw material with a much lower embodied energy than as synthesized PET.

Further work will aim at determining potential applications benefiting from the improved thermo-mechanical behaviour of the blends combined with their excellent environmental impact. In particular, the blends should have higher thermal resistance than neat COPET (composites), at least as measured by the low-weight version of HDT (HDT B), a good indicator of the maximum temperature at which a part is still self-supporting. Hence, everyday non-structural applications requiring heat resistance up to 50–60 °C would be feasible, such as crates, small furniture and even including “hot fill” capability.

## Data Availability

The data presented in this study are available on request from the corresponding author.
